# Cricoid and cervical osteophytes causing dysphagia: an extremely rare and interesting case

**DOI:** 10.1590/0100-3984.2016.0100

**Published:** 2018

**Authors:** Puneet Gupta, Manik Mahajan, Poonam Sharma, Arti Khurana, Indu Bhasin

**Affiliations:** 1 Lady Hardinge Medical College, Delhi University, New Delhi, India.; 2 GMC Hospital, Jammu University, Jammu, J&K, India.; 3 ASCOMS Hospital, Jammu University, Jammu, J&K, India.

**Figure 1:**
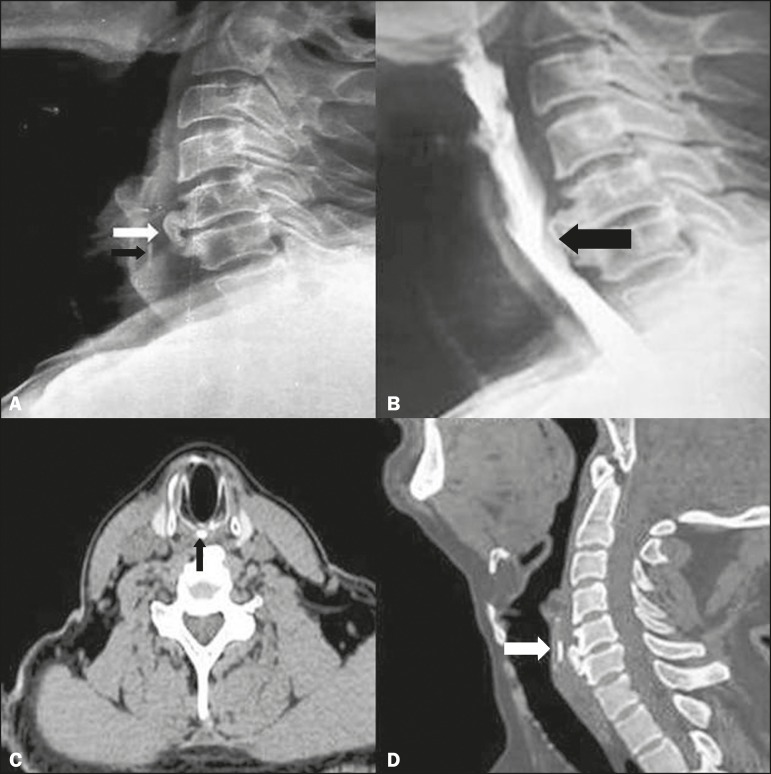
**A:** Soft tissue X-ray of the neck, in lateral view, showing large anterior osteophytes (white arrow) of the C5 and C6 vertebral bodies and an elongated cricoid osteophyte (black arrow). **B:** Barium swallow revealing a smooth extrinsic indentation in the esophagus at the level of the osteophyte formation (arrow). **C:** Axial CT scan showing an osteophyte arising from the cricoid cartilage (arrow). **D:** Sagittal reconstructed CT images showing an elongated cricoid osteophyte caudally (arrow) at the C5-C6 level.

Dear Editor,

A 54-year-old male presented to our department with a twomonth history of nonprogressive dysphagia to solids and irritation in the neck. Physical examination and laboratory findings were unremarkable. Soft tissue X-ray of the neck, in lateral view, revealed anterior bridging osteophytes at the C5-C6 level and an elongated osteophyte in the region of the cricoid cartilage (Figure 1A). An axial computed tomography (CT) scan showed the formation of a spur, 2 mm in diameter, extending from the cricoid cartilage (Figure 1C). Sagittal reconstruction revealed a cricoid osteophyte extending 9 mm caudally at the C5-C6 level (Figure 1D). Barium swallow revealed smooth extrinsic indentation in the esophagus at the level of osteophytes (Figure 1B). The difficulty in swallowing was attributed to the compression of the

There are various pathological causes of dysphagia^1^, including inflammatory diseases of the esophagus, motility dysfunctions, diverticulum, benign or malignant lesions of the mediastinum, cervical spinal diseases, and degenerative diseases of the vertebrae. Although degenerative changes of the cervical spine are frequently seen after the fifth decade of life, dysphagia due to the compression by osteophytes is rare^2^^-^^5^ and dysphagia due to esophageal compression by cricoid osteophytes has rarely been described. Large bridging anterior osteophytes of the cervical spine usually result from diffuse idiopathic skeletal hyperostosis, and the possible explanations for dysphagia include compression of the esophagus, blockage of large osteophytes and osteophyte-induced paraesophageal inflammatory edema. The esophagus is located at the level of the cricoid cartilage, and a small osteophyte at the cricoid cartilage level can therefore cause dysphagia^6^^,^^7^.

Most patients with cervical or thoracic osteophytes indenting the esophagus have no esophageal symptoms. Therefore, osteophytes should be considered as the cause of dysphagia only when other pathologic lesions in the esophagus (e.g., tumors, rings, webs, and strictures) have been excluded. Most osteophytes that induce symptoms are located at the level of the cricoid cartilage. Our case was extremely unusual in that the patient had a cricoid osteophyte as well as large anterior cervical osteophytes, all causing indentation of the esophagus and leading to dysphagia.

The diagnostic imaging approach should include a lateral X-ray, barium swallow, and CT. The formation of an osteophyte, the degree to which it compresses the esophagus, and its extent (craniocaudal and anteroposterior) can be demonstrated by plain lateral radiographs, barium swallow, and CT, respectively^3^. The above mentioned techniques and upper gastrointestinal endoscopy can exclude a neoplasm, a vascular anomaly, and other intrinsic or extrinsic mass lesions, thereby implicating cricoid or cervical osteophytes as a cause of dysphagia, as in our case.

Management of anterior cervical osteophytes can be divided into conservative and surgical methods^8^. Although most patients respond to diet modification, swallowing therapy, nonsteroidal anti-inflammatory drugs, muscle relaxants, antibiotics, and steroids, although surgical treatment is required in severe cases.
